# Impact of ^68^Ga-PSMA PET/CT on salvage radiotherapy planning in patients with prostate cancer and persisting PSA values or biochemical relapse after prostatectomy

**DOI:** 10.1186/s13550-016-0233-4

**Published:** 2016-10-26

**Authors:** Christina Bluemel, Fraenze Linke, Ken Herrmann, Iva Simunovic, Matthias Eiber, Christian Kestler, Andreas K. Buck, Andreas Schirbel, Thorsten A. Bley, Hans-Juergen Wester, Daniel Vergho, Axel Becker

**Affiliations:** 1 Department of Nuclear Medicine, University Hospital Würzburg, Würzburg, Germany; 2 Department of Radiation Oncology, Klinikum Ansbach, Ansbach, Germany; 3 Department of Molecular and Medical Pharmacology, David Geffen School of Medicine at UCLA, Los Angeles, USA; 4 Department of Nuclear Medicine, University Hospital Essen, Essen, Germany; 5 Department of Urology, University Hospital Würzburg, Würzburg, Germany; 6 Department of Diagnostic and Interventional Radiology, University Hospital Würzburg, Würzburg, Germany; 7 Pharmaceutical Radiochemistry, Technische Universität München, Munich, Germany; 8 Department of Nuclear Medicine, University Hospital Würzburg, Oberdürrbacher Str. 6, 97080 Würzburg, Germany

**Keywords:** Prostate cancer, Salvage radiotherapy, PSMA, PET/CT, Recurrence

## Abstract

**Background:**

Salvage radiotherapy (SRT) is clinically established in prostate cancer (PC) patients with PSA persistence or biochemical relapse (BCR) after prior radical surgery. PET/CT imaging prior to SRT may be performed to localize disease recurrence. The recently introduced ^68^Ga-PSMA outperforms other PET tracers for detection of recurrence and is therefore expected also to impact radiation planning.

Forty-five patients with PSA persistence (16 pts) or BCR (29 pts) after prior prostatectomy, scheduled to undergo SRT of the prostate bed, underwent ^68^Ga-PSMA PET/CT. The median PSA level was 0.67 ng/ml. The impact of ^68^Ga-PSMA PET/CT on the treatment decision was assessed. Patients with oligometastatic (≤5 lesions) PC underwent radiotherapy (RT), with the extent of the RT area and dose escalation being based on PET positivity.

**Results:**

Suspicious lesions were detected in 24/45 (53.3 %) patients. In 62.5 % of patients, lesions were only detected by ^68^Ga-PSMA PET. Treatment was changed in 19/45 (42.2 %) patients, e.g., extending SRT to metastases (9/19), administering dose escalation in patients with morphological local recurrence (6/19), or replacing SRT by systemic therapy (2/19). 38/45 (84.4 %) followed the treatment recommendation, with data on clinical follow-up being available in 21 patients treated with SRT. All but one showed biochemical response (mean PSA decline 78 ± 19 %) within a mean follow-up of 8.12 ± 5.23 months.

**Conclusions:**

^68^Ga-PSMA PET/CT impacts treatment planning in more than 40 % of patients scheduled to undergo SRT. Future prospective studies are needed to confirm this significant therapeutic impact on patients prior to SRT.

## Background

Prostate cancer is the most frequent cancer and the third leading cause of death among men in developed countries [1]. Approximately one third to one half of patients suffer from biochemical relapse (BCR) within 5–10 years after primary curative prostatectomy or radiotherapy (RT) [2–4]. Salvage radiotherapy (SRT) is recommended in patients treated with radical prostatectomy who experience BCR without distant metastases [5, 6]. The most evident benefit of SRT has been found in BCR patients with low PSA levels (≤0.5 ng/ml) [7, 8]. Stephenson et al. reported a 6-year post-SRT progression-free probability of 48 % in patients with a PSA <0.5 ng/ml compared with 40, 28, and 18 % in patients with PSA levels of 0.51–1 ng/ml, 1.01–1.5 ng/ml, and >1.5 ng/ml, respectively [9]. The extent of SRT (prostate bed vs. prostate bed and pelvic lymph nodes) is still a matter of controversy [5]. Goldner et al. reported a significantly increased risk for biochemical failure after SRT in patients with a risk of lymph node involvement >15 % according to the Roach formula and SRT limited to the prostate bed [10]. However, automatic inclusion of radiation of the pelvic lymph nodes as part of SRT is currently not recommended [5, 6].

The role of pretherapeutic imaging in SRT planning has not been definitively assessed. The relevance of conventional imaging (e.g., computed tomography, bone scintigraphy) is low due to limited sensitivity in patients with low PSA values [11, 12]. Molecular imaging using choline PET/CT has recently been widely used in patients with BCR. ^11^C-choline PET/CT proved to be sensitive in patients with a PSA level >1 ng/ml and is often used if imaging results are considered relevant for treatment planning [5], e.g., for metastasis-directed therapy in oligometastatic disease [13]. However, the detection rates and accuracy of choline PET/CT are limited [14, 15]. Krause et al. reported a detection rate of <50 % in patients with a PSA level ≤2 ng/ml [16]. A PSA level of 1.16 ng/ml was found to be an optimal cutoff value for prediction of a positive choline PET/CT scan [17]. The American Society for Radiation Oncology (ASTRO) guidelines stated that “improved imaging techniques would help to better define appropriate therapies” [6].

The recent introduction of PET ligands to image the expression of the prostate-specific membrane antigen (PSMA) appears to be revolutionizing prostate cancer imaging and therapy [18, 19]. Numerous studies have reported that ^68^Ga-labeled peptide ligands targeting the cell surface protein PSMA are more specific and also highly sensitive [20–25]. Detection rates of 74.2–89.5 % have been reported in patients with BCR even in the presence of low PSA levels (<1 ng/ml) [20–24]. Van Leeuwen et al. found in a cohort of 70 patients with BCR (PSA level <1.0 ng/ml) and considered for SRT PSMA-positive lesions in 54 % of the patients [26]. A recently published meta-analysis summarized the available studies and reported in patients with BCR an overall positivity rate of 76 % [27]. However, only limited data are available regarding the impact of ^68^Ga-PSMA PET/CT on SRT planning [28–30]. Change of treatment based on the ^68^Ga-PSMA PET/CT findings has been reported in 50.8 and 53.7 % of patients, but these cohorts included primary disease and BCR [29, 30]. Dewes et al. found a change of TNM stage in 53.3 % and a change in RT concept in 33.3 % of cases prior to initial curative RT [28]. Patient cohorts were heterogeneous, and only very limited data are available on patient outcome in ^68^Ga-PSMA PET/CT-guided RT [31]. The aim of this study was to evaluate the impact of ^68^Ga-PSMA PET/CT on treatment decision-making prior to SRT and particularly on the patient outcome.

## Methods

### Patients

Between September 2014 and May 2016, 45 prostate cancer patients with persisting PSA (16/45; 35.6 %) or BCR (29/45; 64.4 %) after radical prostatectomy were referred for a ^68^Ga-PSMA PET/CT prior to SRT (Table 1) to excluded distant metastases. All but one were initially treated with lymph node dissection in addition to prostatectomy. All patients were hormone-naïve at the time of imaging. The mean patient age at the time of initial diagnosis was 63.0 ± 6.9 years (range 46.7–78.8 years) and, at the time of imaging, 68.8 ± 7.0 years (range 52.3–80.0 years). ^68^Ga-PSMA PET/CT was performed 5.7 ± 5.3 years (range 0.15–20.9 years) after initial curative treatment. The mean serum PSA level at the time of ^68^Ga-PSMA PET/CT was 1.30 ± 2.06 ng/ml (range 0.10–11.22 ng/ml; median 0.67 ng/ml). The mean PSA doubling time was 7.4 ± 8.0 months (range 0.0–31.5 months; median 4.8 months).

**Table 1:** Patient characteristics

	No. of patients (%)	Mean (range) (median)
Age at initial diagnosis (years)		63.0 ± 6.9 (46.7–78.8)
Initial Gleason score		7 ± 1 (4–9)
Initial PSA level (ng/ml)		22.46 ± 55.48 (1.85–378.2) (11.2)
Pathologic tumor stage		
T2N0	23 (51)	
T2N1	1 (2)	
T2Nx	1 (2)	
T3N0	8 (18)	
T3N1	9 (20)	
ypT3N0	2 (4)	
ypT3N1	1 (2)	
R status		
R0	30 (67)	
R1	14 (31)	
Rx	1 (2)	
D’Amico risk classification		
High risk	39 (87)	
Intermediate risk	4 (9)	
Low risk	2 (4)	

Due to the retrospective design of this study, a need for formal review was waived by the local ethics committee.

### ^68^Ga-PSMA PET/CT

Images were acquired on a Biograph mCT 64 (Siemens Medical Solutions, Germany). All patients received EuK-Sub-kf(3-iodo-y)-^68^Ga-DOTAGA (^68^Ga-PSMA I&T). ^68^Ga-PSMA I&T was synthesized as previously described [32, 33]. 141 ± 19 MBq (range 97–184 MBq) ^68^Ga-PSMA I&T was injected intravenously in combination with 10–20 mg of furosemide to improve image quality. Images were acquired 60 min after injection from the base of the skull to the proximal thighs (2–3 min/bed position). In 25/45 (55.6 %) patients, contrast-enhanced CT was acquired and also used for attenuation correction. Of the 45 patients, 20 (44.4 %) underwent a low-dose CT protocol due to available contrast-enhanced imaging within 4 weeks prior to ^68^Ga-PSMA PET/CT. Image reconstruction was performed as previously described [22]. PET/CT images were visually analyzed by experienced nuclear medicine physicians and radiologists. In PET, any focal uptake that was not physiological and was higher than the surrounding background was considered suspicious. For PET-positive lesions, the SUV_max_ was measured. For CT image analysis, morphological criteria for malignancy (e.g., small axis diameter of 10 mm for lymph nodes or osteoblastic lesions for bone metastases) were used.

### Treatment decision

All patients were primarily planned to receive SRT of the prostate bed. For the final treatment decision, results of ^68^Ga-PSMA PET/CT were taken into account by an interdisciplinary panel of radiation oncologists, urologists, radiologists, and nuclear medicine physicians. If oligometastatic disease (≤5 metastatic lesions in ≤3 organs) was detected, treatment was changed to high-dose RT of the lesions according to the institutional therapeutic concept, similar to the approach reported in previous publications [13, 29, 31, 34]. In the presence of more than five metastases, the panel recommended systemic treatment with androgen deprivation therapy (ADT).

### Salvage radiotherapy

Image-guided intensity-modulated radiotherapy (IMRT) was performed in all patients receiving RT. Patients were treated on an Elekta Synergy® accelerator using 6-MV photons. SRT was performed in about 7 weeks in five fractions per week. SRT planning was based on CT scans with 3-mm slice thickness, empty rectum, and full bladder. Radiotherapy was carried out in the supine position with daily image guidance with megavoltage cone beam CT (CBCT).

In patients with negative ^68^Ga-PSMA PET/CT, standard RT up to a total dose of 66–70 Gy using a simultaneously integrated boost (SIB) and sequential boost was administered to the prostate bed. Clinical target volume (CTV_prostate bed_) was defined according to the guidelines as described by Poortmans et al. [35]. Pathological tracer uptake in the prostate bed without any morphological correlate on CT resulted in an extension of the CTV_prostate bed_ to this PET-positive area. If a morphological local recurrence was detected on CT, an additional dose escalation up to 76 Gy was administered to the malignant tissue. A 10-mm margin was used to define the planning target volume (PTV) of the prostate bed (PTV_prostate bed_). For the PTV_SIB prostate bed_, the margin was reduced to 5 mm.

When lymph node metastases (LNMs) were evident on CT and/or pathological tracer uptake was seen on PET, an additional CTV_LN_ was defined with a 5-mm margin around the vessels, including the external iliac, internal iliac, and obturator nodes, to a cranial border at the level of the promontory. In the case of pathological retroperitoneal LNMs, the cranial border was extended to a maximum of renal vessels. An additional 5-mm margin was used to define PTV_LN_. Patients received irradiation of the PTV_LN_ at daily doses of 1.8 Gy up to a total of 50.4 Gy. To define the PTV_SIB LN_ for involved nodes, we added a 10-mm margin around the tracer uptake. LNMs detected on ^68^Ga-PSMA PET/CT received a daily SIB of 2 Gy up to 56.0 Gy and a sequential boost up to 60.0–66.0 Gy. The dose was limited by adjacent anatomic structures, e.g., bowel. In accordance with the oligometastatic concept, bone lesions were also irradiated. A total dose of 66.0 Gy was administered. Clinical examination during radiotherapy was done once to twice weekly. Acute side effects were assessed according to the Common Terminology Criteria for Adverse Events (CTCAE 4.0). After the end of SRT, patients were followed up by measurement of serum PSA values to assess the biochemical response to treatment.

### Statistics

Descriptive analysis was performed by calculating the mean, standard deviation (SD), range, and median. PSA doubling time was calculated as previously described [22]. Two-sided two-sample *t* test was used to evaluate changes in PSA levels following therapy, compared with pretherapy levels (significance level *α* = 5 %). Statistical analyses were conducted with Excel statistics software (Excel 2010, Microsoft, WA, USA).

## Results

### ^68^Ga-PSMA PET/CT

^68^Ga-PSMA PET/CT was rated as negative in 21 patients (46.7 %) and positive in 24 (53.3 %). Among these 24 patients, lesions were only detected on ^68^Ga-PSMA PET in 15 (62.5 %) and only on CT in one (4.2 %). In three patients (12.5 %), ^68^Ga-PSMA PET detected more lesions than CT while in the remaining five patients (20.8 %) results from ^68^Ga-PSMA PET and CT were identical. The mean overall SUV_max_ of PET-positive lesions was 16.24 ± 16.97 (range 3.42–93.29), 18.26 ± 14.71 (range 4.22–58.43) in patients receiving low-dose CT, and 13.52 ± 19.67 (range 3.42–93.29) in patients with contrast-enhanced CT. The mean PSA level was 0.75 ± 0.65 ng/ml (range 0.10–2.80 ng/ml; median 0.57 ng/ml) in ^68^Ga-PSMA PET/CT-negative patients and 1.78 ± 2.69 ng/ml (range 0.12–11.22 ng/ml; median 0.70 ng/ml) in ^68^Ga-PSMA PET/CT-positive patients.

Positive ^68^Ga-PSMA PET/CT results were found in all groups according to the D’Amico risk classification. Lesion types and numbers identified on ^68^Ga-PSMA PET/CT are presented in Table 2. Local recurrence was present in 11 of the 45 patients (24.4 %). Eight patients (17.7 %) had only LNMs; one (2.2 %) had local recurrence and pelvic LNMs (<5 lesions) detected only on ^68^Ga-PSMA PET; two (4.4 %) had a local recurrence, pelvic LNM, and bone metastases; and two (4.4 %) had rectal lesions suspicious for metastases.

**Table 2:** Regions involved due to ^68^Ga-PSMA PET/CT

	No. of patients (%)	Suspicious lesions detected by CT, PET, or both (%)			
		Only CT positive	Only PET positive	More lesions in PET	Positive in CT and PET
Negative	21 (46.7)	–	–	–	–
Loc rec	11 (24.4)	1 (9.0)	5 (45.5)	–	5 (45.5)
LNM	8 (17.7)	–	7 (87.5)	1 (12.5)	–
LNMp (<5 lesions)	6				
LNMp + r (<5 lesions)	1				
LNMp + r (>5 lesions)	1				
Loc rec + LNMp (<5 lesions)	1 (2.2)	–	1 (100)	–	–
Loc rec + LNMp + bone	2 (4.4)	–	–	2 (100)	–
< 5 lesions	1				
> 5 lesions	1				
Soft tissue metastasis (rectal)	2 (4.4)	–	2 (100)	–	–

SUV_max_ of PET positive lesions: mean 16.24 ± 16.97 (range 3.42–93.29)

*Loc rec* local recurrence, *LNM* lymph node metastases, *p* pelvic, *r* retoperitoneal, *bone* bone metastases

### Treatment decision

**Fig. 1:**
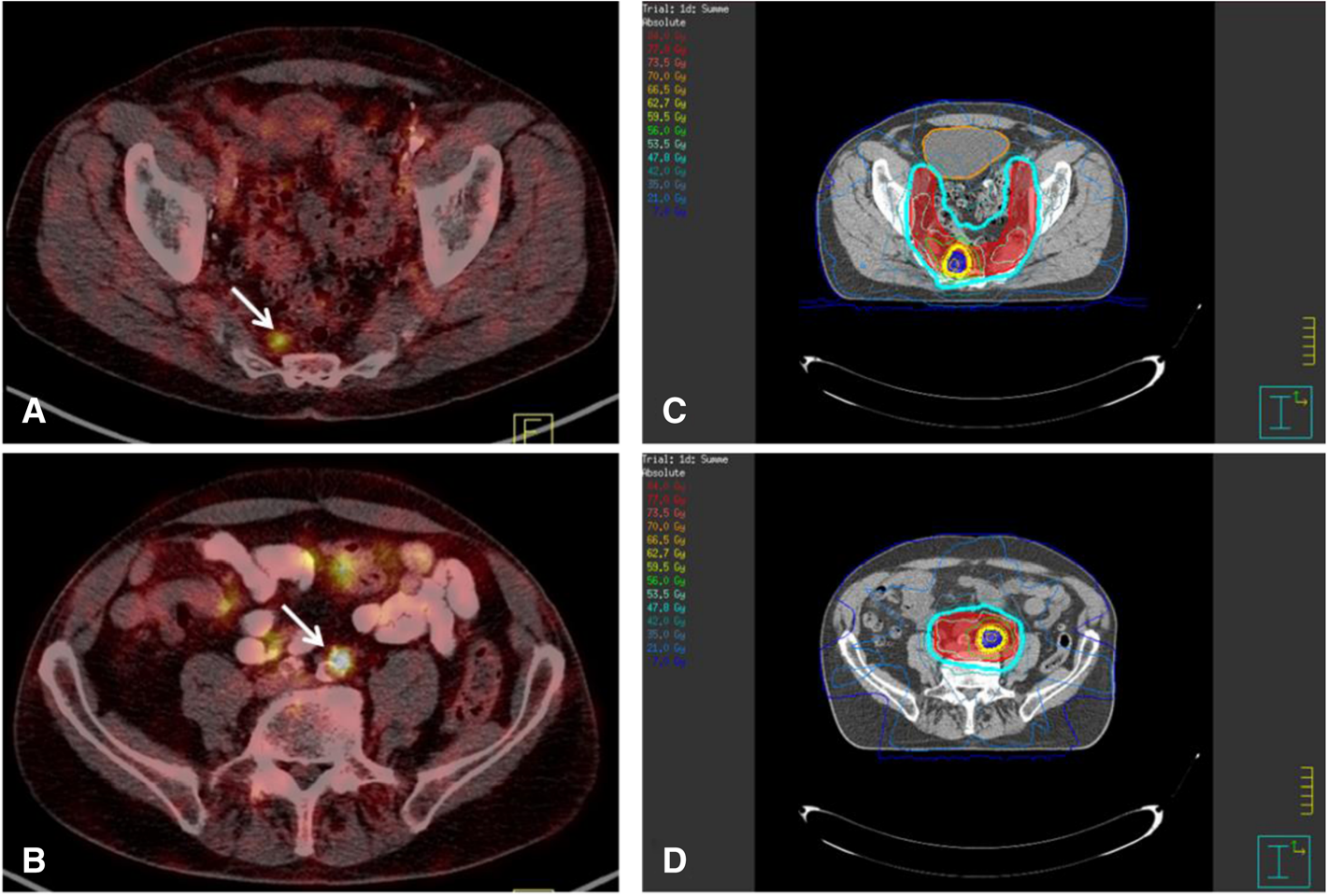
A 74-year-old patient with biochemical recurrence (PSA 0.82 ng/ml; pT2aN0cM0; Gleason 6; iPSA 5.37 ng/ml) 8.4 months after radical prostatectomy and lymph node dissection. ^68^Ga-PSMA PET/CT prior to salvage radiotherapy showed two PSMA-positive presacral (**a**, **c**) and retroperitoneal (**b**, **d**) LNMs. Salvage radiotherapy was extended to pelvic lymph nodes, including a dose escalation to the PSMA-positive lymph nodes. The patient was treated with IMRT (**c**, **d** IMRT plan). *red* PTV including pelvic lymph nodes (50.4 Gy), *blue* simultaneous and sequential boost (66 Gy) for iliac (**d**) and presacral (**c**) LNM. RT to prostate bed is not shown. The PSA level decreased to 0.02 ng/ml after SRT

**Fig. 2:**
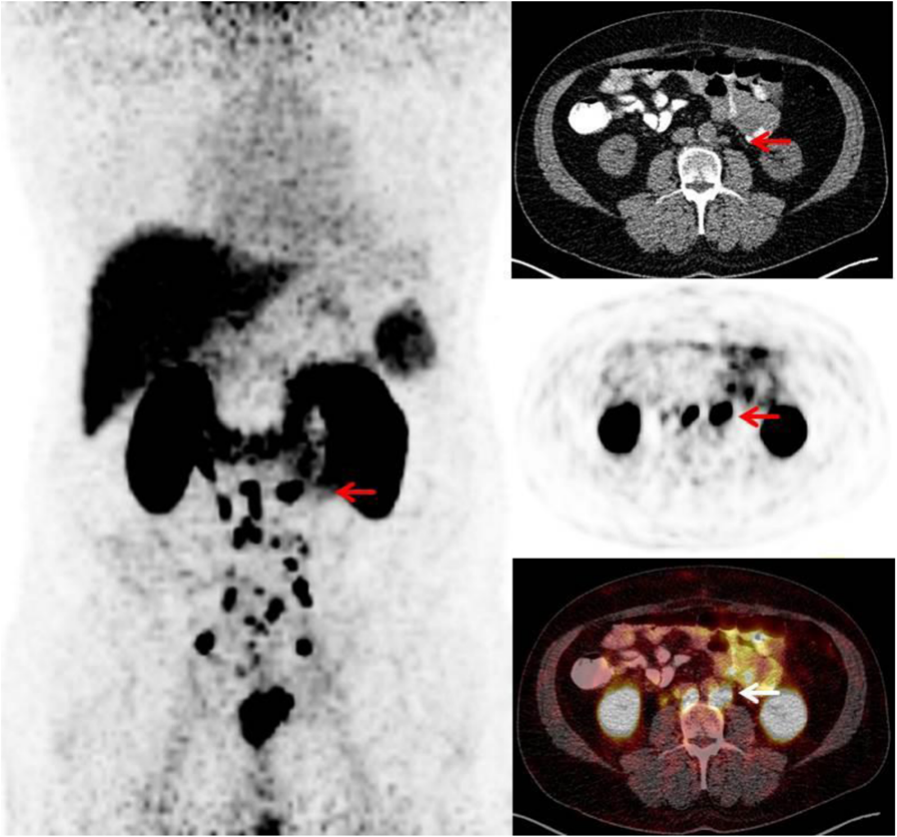
A 62-year-old patient with persisting PSA level (0.7 ng/ml) after radical prostatectomy. Initial PSA level 9.24 ng/ml. High-risk (G3) prostate cancer pT3aN1. ^68^Ga-PSMA PET/CT detected multiple small pelvic and retroperitoneal (*arrow*) LNMs. The intended treatment was changed to ADT

The interdisciplinary panel confirmed the intended SRT of the prostate bed in 26 of the 45 patients (57.8 %) and changed the treatment recommendation in the remaining 19 (42.2 %) due to findings on ^68^Ga-PSMA PET/CT (Table 3). In 6/19 patients (31.6 %), a dose escalation was administered due to morphological local recurrence. One of these patients received brachytherapy to avoid side effects to a previously constructed neo-bladder. In 2/19 (10.5 %) patients, the RT field was extended due to a suspicious rectal lesion (without and in combination with surgical resection). In 8/19 (42.1 %) patients, SRT was extended to pelvic and/or retroperitoneal LNMs including dose escalation for involved lymph nodes (Fig. 1). In 1/19 (5.3 %) patients, SRT was extended to LNMs and a single bone metastasis. In 2/19 (10.5 %) patients with multiple distant metastases (bone) and/or multiple LNMs, the panel recommended systemic treatment with ADT (Fig. 2). No patient undergoing SRT received ADT.

**Table 3:** Treatment recommendations of the panel and follow-up of patients

	No. of patients (*n* = 45)	No. of patients following treatment recommendation (*n* = 38)	No. of patients with follow-up available (*n* = 22)
Confirmed SRT of the prostate bed	26/45	19/26	13/19
Treatment changed	19/45	19/19	9/19
Dose escalation RT	6	6	6
• To mLoc rec	• 5	• 5	• 2
• Brachytherapy of mLoc rec	• 1	• 1	• –
SRT extended to LN, including boost	8	8^b^	5
SRT extended to rectal wall	1	1^c^	–
SRT to LN and single bone metastasis	1	1	–
Multimodal concept^a^	1	1	1
ADT	2	2	1^d^

*mLoc rec* morphological local recurrence, *LN* lymph nodes

^**a**^Surgery of rectal soft tissue metastasis, SRT of prostate bed, and RT to rectal wall metastasis (due to R2 resection of metastasis)

^**b**^Therapy was canceled in two patients due to rising PSA levels during RT

^**c**^Therapy was canceled due to rising PSA levels

^**d**^Not included in follow-up calculations

### Treatment and follow-up

Overall, 15.6 % (7/45) of the patients did not follow the treatment regimen recommended by the panel. These patients were excluded from follow-up. Of the 38 patients who followed the treatment recommendation, two (5.6 %) started with ADT and 36 (94.7 %) received SRT alone (35 pts) or in combination with a multimodal therapeutic concept (1 pt). SRT was performed between September 2014 and June 2016. Five patients have not finished the treatment at the time of data analysis. One patient receiving SRT of the prostate bed and one patient receiving an additional boost to a presacral LNM suffered from gastrointestinal toxicity grade 2. In three patients, SRT had to be terminated due to rising PSA levels despite treatment.

Follow-up was available in 21/36 (58.3 %) patients undergoing the recommended SRT approach and finishing SRT (Table 3). Mean follow-up was 8.12 ± 5.23 months (range 1.15–24.36 months; median 6.92 months) using the latest available PSA level. Mean pretherapeutic PSA level in these patients was 0.92 ± 0.89 ng/ml (range 0.10–3.51 ng/ml; median 0.60 ng/ml) and mean post-therapeutic PSA level, 0.34 ± 0.67 ng/ml (range <0.02–2.42 ng/ml; median 0.08 ng/ml). One patient showed a 140 % increase in the pretherapeutic PSA value during post-therapeutic follow-up. PSA values decreased in 20/21 (95.3 %) patients. Mean PSA decline was 78 ± 19 % (range 40–100 %). Post-RT PSA was significantly lower (*p* < 0.01) than pre-RT PSA levels.

## Discussion

Our data demonstrate that ^68^Ga-PSMA PET/CT impacted treatment in 42.2 % (19/45) of patients.

The concept of automatically combining SRT of the prostate bed with radiation of pelvic lymph nodes as part of SRT is controversial [5, 6, 36]. In the present cohort, seven patients (15.6 %) had LNMs limited to the pelvis and would have potentially benefited from extended SRT. Interestingly, according to clinical risk stratifications for identification of patients with high risk of LNMs [37] and therefore suitable for extended irradiation to the pelvic lymph nodes, only five of these seven patients qualified. Moreover, six patients (13.3 %) scheduled for SRT would not have benefited from radiation to the prostate bed and a general extension of SRT to pelvic lymph nodes as they had extrapelvic disease (rectal soft tissue lesions, retroperitoneal LNMs, and/or bone metastases). These data underline the clinical impact of pretherapeutic ^68^Ga-PSMA PET/CT on selection of the most suitable therapeutic approach.

Up to now, only limited data are available on the impact of ^68^Ga-PSMA PET/CT on RT planning [28–30]. Changes in treatment due to ^68^Ga-PSMA PET/CT prior to RT have been reported in previous studies; however, the reported rates were higher compared with our results [29, 30]. Shakespeare found an alteration of treatment in 53.7 % of patients due to ^68^Ga-PSMA PET/CT performed in addition to conventional imaging [29]. This study included an inhomogeneous group of 54 patients including some with PSA relapse after definitive or post-prostatectomy RT and some in whom curative doses of RT were planned (definitive or post-prostatectomy) [29]. Sterzing et al. reported an alteration in the therapeutic approach in 29 (50.8 %) of a cohort of 57 patients with primary and recurrent prostate cancer [28]. Of these patients, 62.1 % received a boost to pelvic LNMs, while in 27.5 %, the irradiation field was extended to retroperitoneal LNMs and 13.8 % received a systemic treatment [28]. A radiation boost to PET-positive lesions seems to be justified due to the high PPV of ^68^Ga-PSMA PET/CT [28]. However, it is crucial to exclude false-positive interpretations due to PSMA-positive celiac ganglia [38].

Pretherapeutic imaging is gaining importance with the emergence of individualized treatment strategies to replace the use of systemic therapies such as ADT for the treatment of all patients with metastatic disease irrespective of the disease extent, thereby avoiding the latter’s various side effects [29, 31, 39, 40]. In the present study, patients with oligometastatic disease (≤5 lesions) were treated with SRT guided by ^68^Ga-PSMA PET/CT. Recently published studies have limited the number of metastases to ≤3 lesions [34, 40]. A consistent definition of the oligometastatic tumor stage is warranted for future trials to allow for comparison of results. The strategy of metastasis-directed treatment in the management of prostate cancer patients with oligometastatic disease as an individualized therapeutic approach is still controversial. Oligometastatic disease seems to be an intermediate and unique clinical state in prostate cancer [40]. Patients with oligometastatic disease may have a superior survival rate [41, 42] and may benefit from metastasis-directed treatment, with delayed clinical progression and postponement of systemic therapy or even cure [34, 40]. Pretherapeutic imaging with a high accuracy is crucial to the use of these novel salvage strategies. Molecular imaging has been found to outperform conventional imaging for assessment of disease extent in cases of BCR [11, 12, 14, 15]. Picchio et al. reported that ^11^C-choline PET/CT is a valuable tool for guidance of RT of ^11^C-choline-positive LNMs and that PET/CT-guided RT resulted in a high early biochemical response rate [39]. Casamassima et al. reported a 3-year local control rate of 90 % in 25 patients treated with RT for LNMs [43]. However, previous studies and meta-analyses reported that choline PET/CT offers a low detection rate in patients with early BCR [14, 16, 44, 45], i.e., the patient cohort recommended for SRT [5, 7, 8]. ^68^Ga-PSMA PET/CT, on the other hand, has been reported to achieve high detection rates, superior to those of choline PET/CT, even in patients with low PSA levels [20–24]. In addition, it has been found that more lesions with a higher SUV_max_ and tumor-to-background ratio are detected by ^68^Ga-PSMA PET/CT compared with choline PET/CT [21, 46]. Therefore, ^68^Ga-PSMA PET/CT is regarded as the preferred method for guidance of RT, particularly in patients with low PSA values [47].

In our total patient cohort, ^68^Ga-PSMA PET/CT-based treatment resulted in biochemical response in all but one patient. PSA values significantly decreased by 78 % compared with pretherapeutic PSA levels. Five of nine patients were treated with a dose escalation to PET-positive LNMs and/or bone metastases using an IMRT technique (oligometastatic concept); the PSA level decreased on average to 15 % of pretherapeutic values. However, three patients had not completed RT as the PSA level was rising during therapy, indicating disease progression, and in one patient, no follow-up was available. Recently, Henkenberens et al. [31] also reported a significant decrease in PSA values in 29 patients followed up for a similar period and also treated according to an oligometastatic concept. ^68^Ga-PSMA PET/CT seems to be a highly sensitive method for detection of oligometastatic disease and guidance of metastasis-directed treatment; however, the oligometastatic concept has to be investigated in further studies.

The current study has several limitations: First, the analysis is a retrospective analysis of consecutive patients referred for ^68^Ga-PSMA PET/CT prior to SRT. Thus, referral bias cannot be excluded. The highly selected nature of the cohort may be the reason for the relatively low detection rate (53.3 %) compared with previous studies investigating ^68^Ga-PSMA PET/CT in large patient cohorts with BCR (74.2–89.5 %) [20, 23, 24] or prior to radiotherapy (73.5 and 96.6 %) [30, 31]. Second, the number of included patients is low, but it is within the range of or superior to the size of patient populations in previously published studies. Third, to date, no long-term follow-up is available for the patients. Fourth, the reason for the treatment failure with increasing post-therapeutic PSA levels in one patient could not be assessed as the patient refused further imaging. Fifth, no histopathological confirmation was available in the present study; however, only few studies are available with histopathologic correlation of the results of ^68^Ga-PSMA PET/CT [27]. Thus, only limited knowledge about the negative predictive value of ^68^Ga-PSMA PET/CT is available. In the present study, also, patients without any PSMA-positive lesion responded to SRT with decreasing PSA values suggesting small lesions may be missed by ^68^Ga-PSMA PET/CT.

## Conclusions

^68^Ga-PSMA PET/CT impacts treatment planning in more than 40 % of patients scheduled to undergo SRT in the presence of PSA persistence or biochemical relapse after prior radical surgery. Future prospective studies are needed to confirm this significant therapeutic impact on patients prior to SRT.

## Abbreviations

ADT: Androgen deprivation therapy
BCR: Biochemical relapse
CTV: Clinical target volume
IMRT: Image-guided intensity-modulated radiotherapy
LNM: Lymph node metastases
PC: Prostate cancer
PSMA: Prostate-specific membrane antigen
PTV: Planning target volume
RT: Radiotherapy
SIB: Simultaneously integrated boost
SRT: Salvage radiotherapy

## References

[1] Torre LA, Bray F, Siegel RL, Ferlay J, Lortet-Tieulent J, Jemal A (2015). Global cancer statistics, 2012. CA Cancer J Clin.

[2] Uchio EM, Aslan M, Wells CK, Calderone J, Concato J (2010). Impact of biochemical recurrence in prostate cancer among US veterans. Arch Intern Med.

[3] Han M, Partin AW, Zahurak M, Piantadosi S, Epstein JI, Walsh PC (2003). Biochemical (prostate specific antigen) recurrence probability following radical prostatectomy for clinically localized prostate cancer. J Urol.

[4] Spahn M, Weiss C, Bader P (2010). Long-term outcome of patients with high-risk prostate cancer following radical prostatectomy and stage-dependent adjuvant androgen deprivation. Urologia Internationalis.

[5] Heidenreich A, Bastian PJ, Bellmunt J (2014). EAU guidelines on prostate cancer. Part II: treatment of advanced, relapsing, and castration-resistant prostate cancer. Eur Urol.

[6] Valicenti RK, Thompson I, Albertsen P (2013). Adjuvant and salvage radiation therapy after prostatectomy: American Society for Radiation Oncology/American Urological Association guidelines. Int J Radiat Oncol Biol Phys.

[7] Swanson GP, Hussey MA, Tangen CM (2007). Predominant treatment failure in postprostatectomy patients is local: analysis of patterns of treatment failure in SWOG 8794. J Clin Oncol.

[8] Pfister D, Bolla M, Briganti A (2014). Early salvage radiotherapy following radical prostatectomy. Eur Urol.

[9] Stephenson AJ, Scardino PT, Kattan MW (2007). Predicting the outcome of salvage radiation therapy for recurrent prostate cancer after radical prostatectomy. J Clin Oncol.

[10] Goldner G, Dimopoulos J, Potter R (2010). Is the Roach formula predictive for biochemical outcome in prostate cancer patients with minimal residual disease undergoing local radiotherapy after radical prostatectomy?. Radiother Oncol.

[11] Kane CJ, Amling CL, Johnstone PA (2003). Limited value of bone scintigraphy and computed tomography in assessing biochemical failure after radical prostatectomy. Urology.

[12] Hovels AM, Heesakkers RA, Adang EM (2008). The diagnostic accuracy of CT and MRI in the staging of pelvic lymph nodes in patients with prostate cancer: a meta-analysis. Clin Radiol.

[13] Ost P, Bossi A, Decaestecker K (2015). Metastasis-directed therapy of regional and distant recurrences after curative treatment of prostate cancer: a systematic review of the literature. Eur Urol.

[14] Fanti S, Minozzi S, Castellucci P (2016). PET/CT with (11)C-choline for evaluation of prostate cancer patients with biochemical recurrence: meta-analysis and critical review of available data. Eur J Nucl Med Mol Imaging.

[15] Ceci F, Castellucci P, Graziani T (2016). (11)C-Choline PET/CT in castration-resistant prostate cancer patients treated with docetaxel. Eur J Nucl Med Mol Imaging.

[16] Krause BJ, Souvatzoglou M, Tuncel M (2008). The detection rate of [11C]choline-PET/CT depends on the serum PSA-value in patients with biochemical recurrence of prostate cancer. Eur J Nucl Med Mol Imaging.

[17] Graziani T, Ceci F, Castellucci P, et al. C-Choline PET/CT for restaging prostate cancer. Results from 4,426 scans in a single-centre patient series. Eur J Nucl Med Mol Imaging. 2016;43:1971–979. 10.1007/s00259-016-3428-z 27277279

[18] Fendler WP, Bluemel C, Rubello D, Herrmann K (2016). Have we overcome choline PET/CT for early detection of prostate cancer recurrence?. Nuclear Medicine Communications.

[19] Mottaghy FM, Behrendt FF, Verburg FA (2016). (68)Ga-PSMA-HBED-CC PET/CT: where molecular imaging has an edge over morphological imaging. Eur J Nucl Med Mol Imaging.

[20] Afshar-Oromieh A, Avtzi E, Giesel FL (2015). The diagnostic value of PET/CT imaging with the (68)Ga-labelled PSMA ligand HBED-CC in the diagnosis of recurrent prostate cancer. Eur J Nucl Med Mol Imaging.

[21] Afshar-Oromieh A, Zechmann CM, Malcher A (2014). Comparison of PET imaging with a (68)Ga-labelled PSMA ligand and (18)F-choline-based PET/CT for the diagnosis of recurrent prostate cancer. Eur J Nucl Med Mol Imaging.

[22] Bluemel C, Krebs M, Polat B (2016). 68Ga-PSMA-PET/CT in patients with biochemical prostate cancer recurrence and negative 18F-choline-PET/CT. Clin Nucl Med.

[23] Ceci F, Uprimny C, Nilica B (2015). (68)Ga-PSMA PET/CT for restaging recurrent prostate cancer: which factors are associated with PET/CT detection rate?. Eur J Nucl Med Mol Imaging.

[24] Eiber M, Maurer T, Souvatzoglou M (2015). Evaluation of hybrid (6)(8)Ga-PSMA ligand PET/CT in 248 patients with biochemical recurrence after radical prostatectomy. J Nucl Med.

[25] Verburg FA, Pfister D, Heidenreich A (2016). Extent of disease in recurrent prostate cancer determined by [(68)Ga]PSMA-HBED-CC PET/CT in relation to PSA levels, PSA doubling time and Gleason score. Eur J Nucl Med Mol Imaging.

[26] van Leeuwen PJ, Stricker P, Hruby G (2016). (68) Ga-PSMA has a high detection rate of prostate cancer recurrence outside the prostatic fossa in patients being considered for salvage radiation treatment. BJU Int.

[27] Perera M, Papa N, Christidis D, et al. Sensitivity, specificity, and predictors of positive 68ga-prostate-specific membrane antigen positron emission tomography in advanced prostate cancer: a systematic review and meta-analysis. Eur Urol. 2016. Epub. 10.1016/j.eururo.2016.06.021 27363387

[28] Dewes S, Schiller K, Sauter K (2016). Integration of (68)Ga-PSMA-PET imaging in planning of primary definitive radiotherapy in prostate cancer: a retrospective study. Radiation Oncology.

[29] Shakespeare TP (2015). Effect of prostate-specific membrane antigen positron emission tomography on the decision-making of radiation oncologists. Radiation Oncology.

[30] Sterzing F, Kratochwil C, Fiedler H (2016). (68)Ga-PSMA-11 PET/CT: a new technique with high potential for the radiotherapeutic management of prostate cancer patients. Eur J Nucl Med Mol Imaging.

[31] Henkenberens C, von Klot CA, Ross TL (2016). 68Ga-PSMA ligand PET/CT-based radiotherapy in locally recurrent and recurrent oligometastatic prostate cancer: early efficacy after primary therapy. Strahlenther Onkol.

[32] Weineisen M, Simecek J, Schottelius M, Schwaiger M, Wester HJ (2014). Synthesis and preclinical evaluation of DOTAGA-conjugated PSMA ligands for functional imaging and endoradiotherapy of prostate cancer. Ejnmmi Research.

[33] Herrmann K, Bluemel C, Weineisen M (2015). Biodistribution and radiation dosimetry for a probe targeting prostate-specific membrane antigen for imaging and therapy. J Nucl Med.

[34] Ost P, Jereczek-Fossa BA, As NV (2016). Progression-free survival following stereotactic body radiotherapy for oligometastatic prostate cancer treatment-naive recurrence: a multi-institutional analysis. Eur Urol.

[35] Poortmans P, Bossi A, Vandeputte K (2007). Guidelines for target volume definition in post-operative radiotherapy for prostate cancer, on behalf of the EORTC Radiation Oncology Group. Radiother Oncol.

[36] Interdisziplinäre Leitlinie der Qualität S3 zur Früherkennung, Diagnose und Therapie der verschiedenen Stadien des Prostatakarzinoms. Langversion 3.1 - 2. Aktualisierung - AWMF-Register-Nummer 043/022OL. 2014.

[37] Spiotto MT, Hancock SL, King CR (2007). Radiotherapy after prostatectomy: improved biochemical relapse-free survival with whole pelvic compared with prostate bed only for high-risk patients. Int J Radiat Oncol Biol Phys.

[38] Krohn T, Verburg FA, Pufe T (2015). [(68)Ga]PSMA-HBED uptake mimicking lymph node metastasis in coeliac ganglia: an important pitfall in clinical practice. Eur J Nucl Med Mol Imaging.

[39] Picchio M, Berardi G, Fodor A (2014). (11)C-Choline PET/CT as a guide to radiation treatment planning of lymph-node relapses in prostate cancer patients. Eur J Nucl Med Mol Imaging.

[40] Bernard B, Gershman B, Karnes RJ, Sweeney CJ, Vapiwala N (2016). Approach to oligometastatic prostate cancer. Am Soc Clin Oncol Educ Book..

[41] Singh D, Yi WS, Brasacchio RA (2004). Is there a favorable subset of patients with prostate cancer who develop oligometastases?. Int J Radiat Oncol Biol Phys.

[42] Abdollah F, Briganti A, Montorsi F (2015). Contemporary role of salvage lymphadenectomy in patients with recurrence following radical prostatectomy. Eur Urol.

[43] Casamassima F, Masi L, Menichelli C (2011). Efficacy of eradicative radiotherapy for limited nodal metastases detected with choline PET scan in prostate cancer patients. Tumori.

[44] Evangelista L, Zattoni F, Guttilla A, Saladini G, Colletti PM, Rubello D (2013). Choline PET or PET/CT and biochemical relapse of prostate cancer: a systematic review and meta-analysis. Clinical Nuclear Medicine.

[45] Umbehr MH, Muntener M, Hany T, Sulser T, Bachmann LM (2013). The role of 11C-choline and 18F-fluorocholine positron emission tomography (PET) and PET/CT in prostate cancer: a systematic review and meta-analysis. Eur Urol.

[46] Morigi JJ, Stricker PD, van Leeuwen PJ (2015). Prospective comparison of 18F-fluoromethylcholine versus 68Ga-PSMA PET/CT in prostate cancer patients who have rising PSA after curative treatment and are being considered for targeted therapy. J Nucl Med.

[47] Evangelista L, Briganti A, Fanti S, et al. New clinical indications for F/C-choline, new tracers for positron emission tomography and a promising hybrid device for prostate cancer staging: a systematic review of the literature. Eur Urol. 2016;70:161–75. 10.1016/j.eururo.2016.01.029 26850970

